# Low intensity blood parasite infections do not reduce the aerobic performance of migratory birds

**DOI:** 10.1098/rspb.2017.2307

**Published:** 2018-01-31

**Authors:** Steffen Hahn, Silke Bauer, Dimitar Dimitrov, Tamara Emmenegger, Karina Ivanova, Pavel Zehtindjiev, William A. Buttemer

**Affiliations:** 1 Department of Bird Migration, Swiss Ornithological Institute, Seerose 1, 6204 Sempach, Switzerland; 2 Institute of Biodiversity and Ecosystem Research, Bulgarian Academy of Sciences, 2 Gagarin Street, Sofia 1113, Bulgaria; 3 School of Biological Sciences, University of Wollongong, Wollongong, New South Wales 2522, Australia

**Keywords:** avian malaria, disease, migration, metabolic rate, oxygen consumption, pathogen

## Abstract

Blood parasites (Haemosporidia) are thought to impair the flight performance of infected animals, and therefore, infected birds are expected to differ from their non-infected counterparts in migratory capacity. Since haemosporidians invade host erythrocytes, it is commonly assumed that infected individuals will have compromised aerobic capacity, but this has not been examined in free-living birds. We tested if haemosporidian infections affect aerobic performance by examining metabolic rates and exercise endurance in migratory great reed warblers (*Acrocephalus arundinaceus*) experimentally treated with *Plasmodium relictum* pGRW04 and in naturally infected wild birds over consecutive life-history stages. We found no effect of acute or chronic infections on resting metabolic rate, maximum metabolic rate or exercise endurance in either experimentally treated or free-living birds. Oxygen consumption rates during rest and while undergoing maximum exercise as well as exercise endurance increased from breeding to migration stages in both infected and non-infected birds. Importantly, phenotypic changes associated with preparation for migration were similarly unaffected by parasitaemia. Consequently, migratory birds experiencing parasitaemia levels typical of chronic infection do not differ in migratory capacity from their uninfected counterparts. Thus, if infected hosts differ from uninfected conspecifics in migration phenology, other mechanisms besides aerobic capacity should be considered.

## 1. Introduction

Parasites are thought to adversely affect the physical performance of their hosts [1] and to be key factors in shaping life history, with effects cascading to communities and ecosystems [2]. Haemosporidian parasites, the causative agents of malaria in the broader sense, commonly occur in temperate and tropical regions and infect a variety of host species including humans, other mammals, reptiles and birds. Depending on lineage and host species, the parasites invade their hosts' inner organs and, particularly, red blood cells [3]. The infection follows a typical pattern with an acute (symptomatic) phase with high levels of parasites in the blood (parasitaemia) followed by a chronic phase with low (or zero) parasitaemia [3]. Malaria in wildlife is often a mild disease, but can have severe consequences, particularly when encountered in new environments by immunologically naive hosts [4]. Acute infections (with high parasitaemia) can result in substantial declines in red blood cell content [5] owing to haemosporidians targeting haemoglobin as a major nutrient [6].

Given the very high rates of oxygen delivery required by birds to sustain the aerobic demands for flapping flight, it is somewhat perplexing that even successful long-distance migrants commonly display chronic malaria infections [4,7]. Although parasitaemia in chronically infected birds is substantially lower than during a primary infection [8], the consequences of low parasitaemia on the aerobic metabolism of birds have not been examined. Measuring the aerobic metabolism can provide important insights into two potential effects of chronic parasitaemia: the energetic cost of a persistent infection through measurements of resting metabolic rate (RMR) and its consequences on the aerobic performance of the infected individual through measurements of maximum metabolic rate (MMR) [9,10].

Endothermic animals have a basal, or minimal, rate of metabolism (basal metabolic rate; BMR) when they are post-absorptive, asleep in the rest-phase of their daily cycle, exposed to thermoneutral temperatures, and not engaged in energetically demanding life-history stages. Thus, the BMR represents an individual's baseline costs of maintaining vital functions [11]. Metabolic measurements made under the same conditions but at times associated with elevated maintenance costs, such as during moult, during the day, or at temperatures outside the thermoneutral zone are termed RMR. Thus, BMR and RMR are pertinent reflections of the energetic costs of physiological processes at particular life-history stages and, thus, can be used to quantify the contemporaneous energy costs of parasite infections. Surprisingly, studies examining the effects of endoparasitic or ectoparasitic infection on host RMR are inconsistent, finding lower, higher or unchanged RMR owing to parasitic infections (reviewed in [12]). However, even if infections have minimal effect on an animal's maintenance metabolism, they could still limit their host's capacity for intense aerobic activities [9]. Reduced aerobic capacity would be especially deleterious for long-distance flyers, as such flight requires sustained delivery and uptake of oxygen at high rates [13].

The potential for parasite infections to affect aerobic capacity can be determined by measuring the MMR. The highest rates of aerobic metabolism in endotherms are associated with sustainable exercise [14]; consequently, MMR can only be measured when animals attain peak levels of locomotor activities. In addition, because MMR depends on the integrated performance of body functions ranging from enzymes to organ systems [15], its measurement may provide insight into an animal's overall physiological vigour.

Many studies have measured RMRs of birds and their variation with body size, phylogeny, season and life-history stage [10]. Little is known, however, about sources of variation in MMR and virtually nothing about the effects of parasite infections on MMR.

We have examined the metabolic consequences of blood parasite infections over three consecutive stages of the annual cycle in great reed warblers (*Acrocephalus arundinaceus*), which are long-distance Palaearctic migrants with non-breeding sites in tropical Africa [16]. We expect a haemosporidian infection will decrease a host's aerobic capacity, and, in turn, reduce both its MMR and its exercise endurance. Moreover, we expect that adverse effects of parasite infection to be particularly apparent during the migration stage, with its associated requirement for high aerobic capacity.

To this end, we measured RMR and MMR in infected and uninfected birds from late breeding until early migration. Specifically, we employed a two-tiered approach: (i) we experimentally inoculated captive, previously uninfected individuals with a parasite strain selected for low virulence and monitored the developing infections along with repeated measures of aerobic performance for comparison with that of uninfected controls; and (ii) we undertook the same physiological appraisal of freshly captured birds, knowing they were likely to have a higher variety of infections with naturally occurring haemosporidian parasites and a greater range of parasitaemia than our captive birds. As an additional performance measure, we recorded the duration of sustained intense exercise during individual MMR measurements.

## 2. Material and methods

The study was carried out on a population of great reed warblers (*A. arundinaceus*) breeding in the Danube River floodplains near Kalimok Biological station (44°00′ N, 26°26′ E, Bulgaria). This population is naturally infected with haemosporidian parasites with a prevalence of approximately 27–40% [17–19].

Great reed warblers were caught in reed beds using mist nets from April to August 2015. Birds were measured (body mass and wing length), aged, ringed and sexed. Additionally, we sampled approx. 30–50 µl blood from a brachial vein for blood smears, measured haemoglobin content (mg ml^–1^) using a HemoCue Hb201, and the remainder was stored in 0.5–1 ml SET-buffer (0.15 M NaCl, 0.01 M Tris, 0.001 M EDTA, pH 8.0) for subsequent genetic analyses of parasite types.

We analysed the Giemsa stained blood smears by microscopy to quantify parasitaemia as the proportion of infected red blood cells (%). This was determined by counting infected erythrocytes in 100 randomly selected microscopic fields under 1000 magnification and by relating the number of infected to the total number of erythrocytes per microscopic field, determined from five pictures taken every 20 fields (mean number of erythrocytes per microscopic field: 306 ± 69 (s.d.), *n* = 550).

For the genetic analyses, we extracted total DNA from blood samples and tested for *Plasmodium* and *Haemoproteus* infections by nested PCR protocol for mitochondrial cyt *b* [20]. Positive samples were sequenced by Macrogen Inc. (http://www.macrogen.com) to discriminate between *Plasmodium* and *Haemoproteus*.

### (a) Measuring aerobic performance

We quantified oxygen consumption rates during overnight rest (RMR), and during forced exercise (MMR) using flow-through respirometry. The aerobic performance was measured from July until the end of August, which includes late-breeding, post-breeding and the initial autumn migration periods of great reed warblers [21]. Because birds had just completed breeding or were in pre-migratory or migratory stages, the strict conditions of measuring BMR might not be fully met and, thus, we consider overnight oxygen consumption rates to represent RMRs [22,23].

We defined RMR (V̇O_2_) as the lowest oxygen consumption rate over a 5 min period during nocturnal rest under thermoneutral conditions (30°C) and in a post-absorptive state. For measurements, birds were placed in individual respirometry chambers (4 l volume; for details see [24,25]). Measurements started around 21.00 h and lasted until the following morning at 06.00 h. Oxygen consumption rate (ml min^–1^) was determined from differences between inlet and outlet O_2_ concentrations by an Oxzilla II Differential Oxygen Analyzer (Sable Systems, NV, USA). Gas flow rate was regulated at 500 ml min^–1^ using calibrated mass-flow metres (Tylan Corp.) and all oxygen measurements were baselined using reference air at 30-min intervals. Data were analysed using LabAnalyst software (http://warthog.ucr.edu).

MMR (V̇O_2_ max) were determined from oxygen consumption during exercise in a hop-flutter wheel, where birds were encouraged to repeatedly take off [10,24]. The effective volume of the MMR system was 36.0 l, with air supplied at a rate of 7.5 l min^−1^ using a calibrated mass-flow controller (MKS Instruments). The rotation speed of the wheel was manually adjusted to each bird's behaviour and fully stopped when the bird could not hold its position in the wheel (which was recorded as time until exhaustion, min). Oxygen consumption during exercise was continuously recorded with an Oxzilla II differential oxygen analyser or an FC1 oxygen analyser (all Sable Systems, NV, USA), using inlet air as a reference at the start and end of each measurement. The maximum VO_2_ was computed from the highest instantaneous oxygen consumption values measured over a 30 s interval of exercise, after baseline correction and smoothing the data to remove electrical noise (1 s smoothing interval over three cycles). All data were processed using LabHelper and LabAnalyst (http://warthog.ucr.edu) software to obtain oxygen consumption rates.

### (b) Experimental infection

Prior to the actual infection experiment, we needed to select a specific parasite strain for experimental infection and constitute donors for both experimental and control groups. We chose *Plasmodium relictum* cyt *b* lineage pGRW04, which naturally occurs in our study species [26]. From catches of wild birds in spring 2015, we selected one individual with a natural pGRW04 infection and five non-infected individuals. All birds were kept indoors in isolated vector-proof cages (100 × 60 × 45 cm) supplied with water and food ad libitum (living mealworms plus a mix of boiled eggs and commercial dry food for insectivorous birds (www.versele-laga.com). The pGRW04 infection was multiplied in four recipient birds by sub-inoculating infected blood into the pectoral muscle. We injected approximately 250 µl of a blood mixture (infected blood, 3.7% sodium citrate solution and 0.9% sodium chloride solution in a 4:1:1 mixture) into each recipient's breast muscle within 5 min after blood withdrawal. One non-infected individual was spared and used as a donor for the control group (see below).

For the infection experiment, we caught wild birds at the end of the breeding season in June and July and selected adult males to minimize variation owing to sex or age. All individuals were evaluated for infection, with those testing negative quarantined for at least one week before confirming infection status. These birds were then kept in vector-protected aviaries furnished with cut reeds, several feeders and water ad libitum. The aviaries allowed free ranging between an indoor (1.3–2.6 × 2.3 × 2.5 m) and an outdoor section (size: 1.5–2.5 × 1.1 × 2.4 m).

Thirty-two individuals entered the experiment and were randomly assigned to either a ‘control’ or ‘experimental’ group. The 16 experimental individuals received blood from the infected donor group and the 16 controls received blood from a non-infected donor, following the above procedure. The experimental protocol generally followed [27].

Starting 5 days after experimental or control inoculation, we determined infection status of each bird from microscopic examination of blood smears collected at 3 day intervals for at least 25 more days or when parasitaemia had dropped to a chronic level (median: 0.0015%).

All experimental birds were successfully released at the end of the study.

### (c) Natural infection

In addition to the infection experiment, we examined 124 free-living great reed warblers (95 males, 12 females and 17 birds of unknown sex) over late-breeding (July 2015), post-breeding (late July/early August) and migration periods (late August). In addition to standard biometric measurements of body mass, fat score, pectoral muscle score and wing size [28], we determined each bird's infection status and parasitaemia by PCR and microscopy. The total haemosporidian prevalence in the sample for the seasonal effect measurement was 45.2% with *Plasmodium* ssp. infections accounting for 13.0%, *Haemoproteus* ssp. for 29.9% and mixed infections for 2.6%. In nine birds, the PCR signal was positive yet no infected cells were found by microscopy. Therefore, parasitaemia must have been lower than one infected cell in 100 microscopic fields (less than 0.003%) and we set it to 0.0015%, i.e. half of the value at microscopic detection limit. Finally, for the later analyses, we categorized birds according to their parasitaemia with zero, low (less than 0.2%) and high parasitaemia (greater than 0.2 to max. 4.0%). The ‘high-parasitaemia’ category is mainly formed by hosts with a *Haemoproteus* infection, although these levels are much lower than those generally associated with acute primary infections [29]. In total, the free-living bird samples contained 54.5% non-infected, 29.2% low parasitaemia and 16.2% high-parasitaemia individuals (*n* = 156 birds incl. experimental birds before infection).

### (d) Metabolic rate measurements

We followed the same experimental protocols throughout all metabolic measurements: (i) we placed birds in holding cages with access to water but no food for 3 h before starting the RMR measurements, (ii) we then placed them in respirometers within a constant-temperature cabinet and measured RMR overnight, (iii) birds were returned to holding cages the next morning and given free access to food and water for about 3–4 h, (iv) we then measured their MMR and, lastly (v) we took a blood sample at completion of MMR to determine each bird's infection status, parasitaemia and haemoglobin concentration.

By contrast to naturally infected birds, which were only measured once, birds of the experimental groups were measured repeatedly: an initial measurement made on average 9 days before inoculation (range: 4–16 days), a second measurement during the acute-infection stage (approx. 23 days post inoculation; range: 14–34 days) and finally, during the chronic infection stage (approx. 34 days post inoculation; range 31–41 days).

### (e) Statistical analyses

To examine the consequences of infections on metabolic rates, we tested whether RMR or MMR changed with infections and/or parasitaemia. In addition to metabolic rates, we also considered other variables that may relate to aerobic performance: the time to exhaustion, and haemoglobin content of whole blood. As metabolic rates scale with body mass and often change seasonally, we included body mass and time of the season as covariates in the models. Data of metabolic rates, body mass, haemoglobin content and time to exhaustion were log_10_ transformed before analyses.

The statistical approaches and general models were equivalent for experimental and natural infections, with a few exceptions: we used linear models to test how the above output measures changed with infection status, time of season/experimental stage and body mass. As individuals were repeatedly measured in the infection experiment and thus, data were non-independent, we used linear mixed effects models, including bird-ID as random factor for the experimental infections and simple linear models for the natural infections.

Specifically for the experiment, the models related RMR, MMR and the other output measures to the interaction of experimental group (‘experimental’ and ‘control’) and phases of the experiment (‘before’ experimental infection, during ‘acute’ or ‘chronic’ phase of infection), while controlling for repeated measurements. We applied Bayesian simulation techniques to compute posterior probabilities for differences between experimental groups. All analyses were run in R (v. 3.3.2) [30], using the R-packages ‘arm’ (v. 1.9–3) [31] and ‘lme4’ (v. 1.1-12) [32]. As the lmer function does not provide *p*-values, we considered factors with absolute *t*-values greater than 2 significant at the *p* < 0.05 level following suggestions in [33].

## 3. Results

### (a) Parasitaemia of blood parasite infection

**Figure 1.**
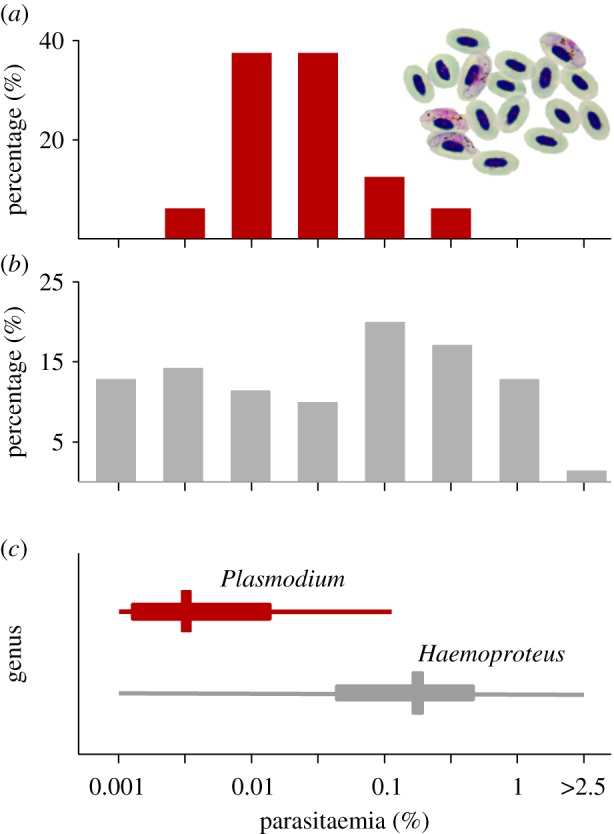
Parasitaemia in experimentally treated and naturally infected great reed warblers. (*a*) Frequency of parasitaemia of the lineage pGRW04 induced in the experimental birds during the acute phase of infection (*n* = 16) and (*b*) total frequency of parasitaemia in chronically infected wild birds (*n* = 61). (*c*) Genus-specific parasitaemia (medians ± 25/75% and the ranges) in wild birds for *Plasmodium* ssp*.* (red, *n* = 18) and *Haemoproteus* ssp*.* (dark grey, *n* = 40).

The median parasitaemia in experimental birds was zero before infection, peaked to 0.030% (range: 0.004–0.548%) during the acute phase and dropped to 0.007% during the chronic phase (range: 0–0.038%) (figure 1*a*). Parasitaemia in the control group remained zero throughout.

By contrast, parasitaemia in freshly captured wild birds averaged 0.103% (figure 1*b*), with *Plasmodium* infections having lower median parasitaemia (0.006%) than *Haemoproteus* infections (0.239%) (figure 1*c*). Thus, naturally infected birds showed a greater range of parasitaemia, and had parasitaemia levels about twofold higher than experimentally infected birds during their acute-infection stage.

### (b) Metabolic rates and endurance

#### (i) Resting metabolic rates

**Figure 2.**
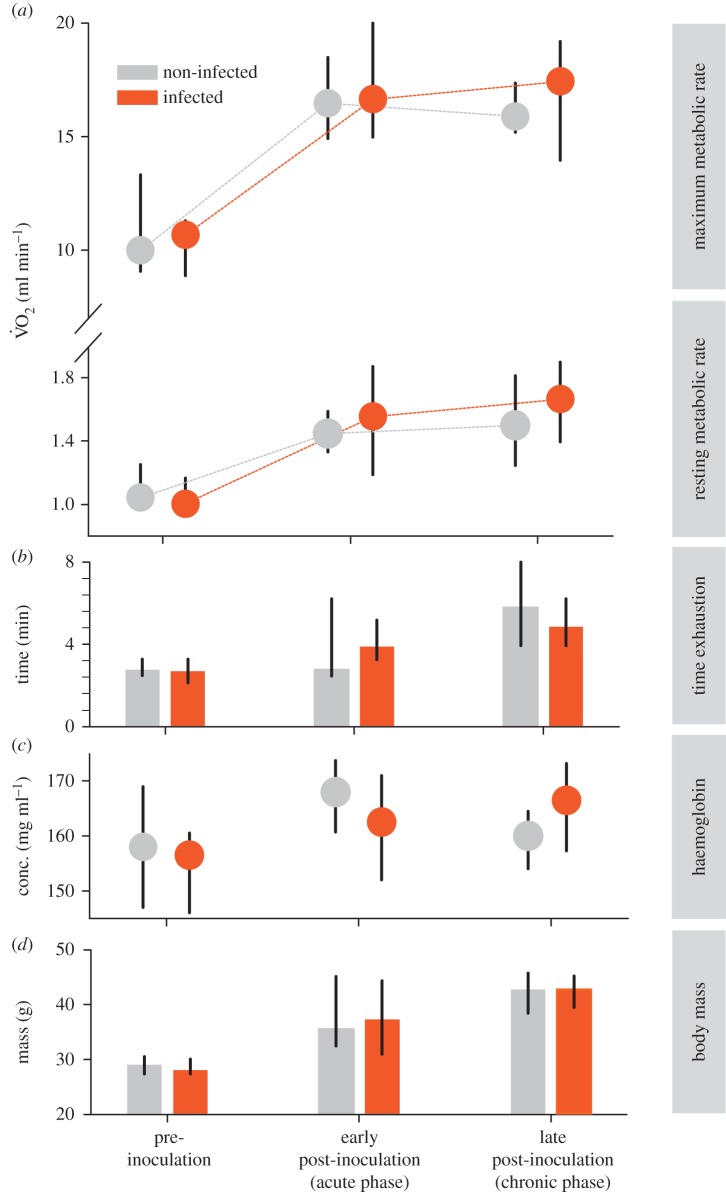
Aerobic performance, haemoglobin content, and body mass of experimentally infected and sham-treated (non-infected) great reed warblers during sequential phases of infection with *Plasmodium relictum*. The aerobic performance is described as oxygen consumption (V̇O_2_, in ml min^−1^) during overnight rest (RMR), and during strenuous exercise (MMR), as well as the endurance of exercise period (time until exhaustion, min). Infected individuals during acute and chronic infection phases (early and late post-inoculation) did not differ from the control group, with all birds showing increasing whole-body metabolic rates and haemoglobin concentrations during the course of the experiment. Data are medians ± 25/75%.

Whole-body RMR in captive birds was much lower before inoculation than at both early and late post-inoculation stages (figure 2), but did not differ between experimentally infected and control individuals at any stage of measurement (figure 2; electronic supplementary material, table S1).

**Figure 3.**
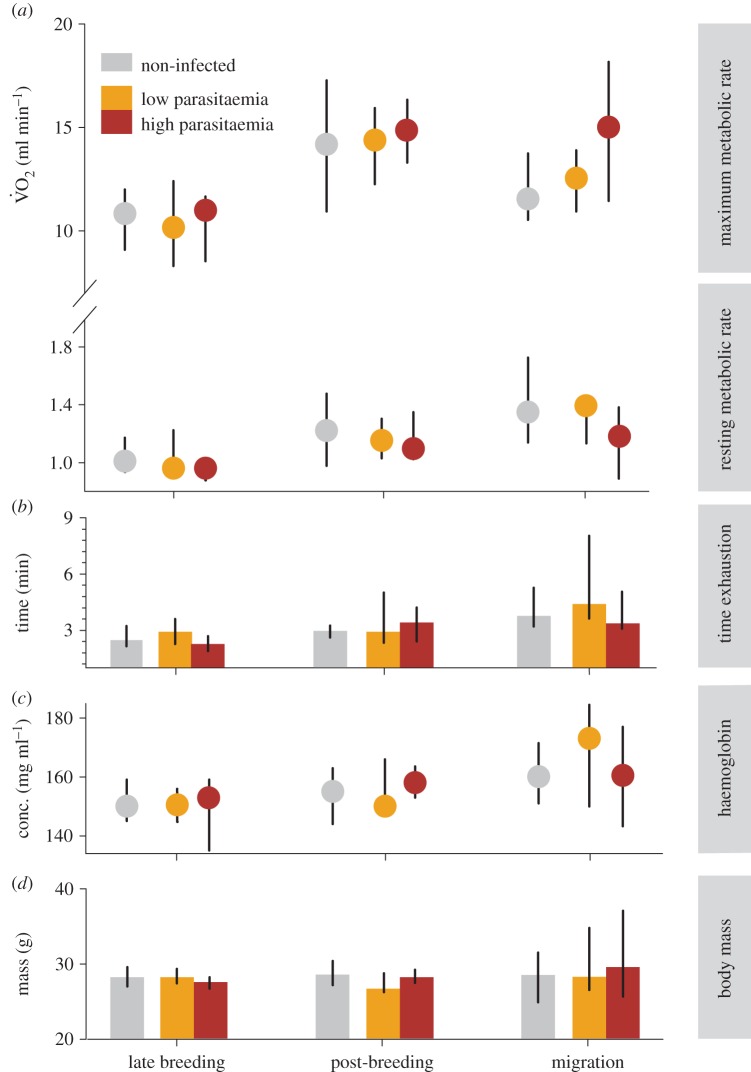
Aerobic performance, haemoglobin concentration and body mass of free-living non-infected and chronically infected great reed warblers during sequential stages of the annual cycle. Infected birds were subdivided into low- and high-parasitaemia birds. Infection status had no effect on aerobic performance, haemoglobin content or period of exercise endurance during any stage we sampled. Thus, birds with chronic low and high parasitaemia were as aerobically capable as their non-infected counterparts, even during the migration phase. Data are medians ± 25/75%.

In wild-caught birds, RMR increased significantly from late-breeding to both post-breeding and migration periods (*p* < 0.05 and 0.01, respectively; figure 3). Within all stages, RMR of non-infected birds was indistinguishable from those with low parasitaemia (*t* = −1.40, *p* = 0.27); however, birds with high parasitaemia had significantly lower RMR during the migration period compared to non-infected birds (*t* = −2.11, *p* = 0.04).

#### (ii) Maximum metabolic rates

Surprisingly, MMR did not differ between experimentally infected and control birds at any phase of the experiment (*t* = −0.20; figure 2), nor did it differ between free-living birds with zero, low or high parasitaemia at any period (*p* > 0.47; figure 3; electronic supplementary material, table S2). However, MMR changed greatly over the study period in both captive and wild birds. In experimental birds, MMR increased by about 50% from pre-inoculation to early post-inoculation phases and remained high until the end of the experiment (figure 2). Although these rises in MMR paralleled increases in body mass in captive birds (figure 2), MMR increased relatively more than mass and much of this mass-increase was associated with fat accumulation (electronic supplementary material, figure S1), which has negligible metabolic activity.

In wild-caught birds, MMR increased by 34% from the late-breeding to post-breeding periods, but was only 16% higher during the migration than late-breeding periods (figure 3).

Notably, the MMR of birds with haemosporidian infections did not differ from that of uninfected birds at any stage of measurement.

#### (iii) Exercise endurance

The time to exhaustion increased over the experimental period from an average of 2.8 to 6.1 min in captive birds, but was not different between infected and non-infected cohorts (*t* = −0.42; figure 2; electronic supplementary material, table S1). In wild-caught individuals, the time to exhaustion also increased significantly over the three life-history stages (*p* < 0.01), averaging about 30% and 65% higher during post-breeding and migration, respectively, than during the late-breeding stages (figure 3). Endurance times of infected free-living birds did not differ from their parasite-free conspecifics during any period of measurement (low parasitaemia: *t* = 1.42, high parasitaemia: *t* = −0.91). Thus, birds in migratory disposition could sustain energy-demanding exercise for much longer than at earlier phases, irrespective of parasitaemia.

### (c) Changes in phenotype

#### (i) Morphology

All captive birds in the experiment gained substantial mass over the course of the study, with an increase of about 46% from the pre-inoculation to the late post-inoculation periods (figure 2). The visible subcutaneous fat stores of these birds increased significantly from pre-inoculation until the early post-inoculation period (*t* = 7.71) and increased somewhat further during the late post-inoculation period (electronic supplementary material, figure S1 and table S1). The pectoral muscle size also increased over these periods, but this muscle score only statistically differed between late post-inoculation and the pre-inoculation stages (*t* = 3.46).

Free-living birds also increased body mass over the study period, but not to the same extent as in captives (figure 3). Fat scores also increased in free-living birds over the course of our measurements, but never reached the levels of captive birds that had access to food ad libitum (electronic supplementary material, figure S2). Pectoral muscle scores were less variable between stages in free-living birds, but were higher during the migration stage (*t* = 3.23, *p* = 0.002; electronic supplementary material, table S2). Importantly, parasitaemia had no effect on any of these morphological variables at any stage we sampled (electronic supplementary material, tables S1 and S2).

#### (ii) Blood haemoglobin concentration

Similar to the lack of effect of haemosporidian infection on aerobic performance, haemoglobin concentration did not differ between infected and non-infected captive birds (*t* = −1.21; figure 2), indicating minimal damage to haemoglobin at the low-parasitaemia levels that *Plasmodium* strain pGRW04 elicited in great reed warblers. Similarly, haemoglobin concentration did not differ between non-infected free-living birds and those with either low or high parasitaemia over the course of our study (both *p* > 0.95; figure 3). By contrast, haemoglobin concentration increased significantly from late-breeding to subsequent periods (*p* < 0.05 and 0.001 for post-breeding and migration periods, respectively), reaching levels about 11% higher during migration compared with late-breeding stages (figure 3).

## 4. Discussion

Our study provides empirical and experimental evidence that aerobic performance is not adversely affected in malaria-infected birds with low parasitaemia. Furthermore, chronically infected birds did not differ from their uninfected counterparts in phenotypic attributes associated with preparations for migration, such as accumulation of lipid stores, increased body mass and higher total haemoglobin concentration in blood [34,35]. Consequently, birds with low parasitaemia, which typifies birds with chronic infections, appear to have the same migratory capacity as uninfected birds.

We derived our experimental results from a well-established host–parasite pair: the *P. relictum* lineage pGRW04 is a host generalist (recorded in 75 host species of 22 bird families around the globe, http://mbio-serv2.mbioekol.lu.se/Malavi/ (accessed 14 September 2017)) and has been frequently found in great reed warbler-breeding populations in Europe [19,26,36] as well as non-breeding populations in Africa [37]. We are confident that the results of our study are robust because both the experimental manipulation as well as the observations on wild, naturally infected adults with higher parasitaemia by *Plasmodium* and *Haemoproteus* showed the same pattern in metabolic rates (see below).

### (a) Metabolic rates and infection

Our results consistently showed that low parasitaemia during the acute or chronic phases of infection did not alter the maintenance metabolism, suggesting that the immune defences in low-parasitaemia settings are energetically frugal, unlike the significant rises in RMR displayed by strong immune responses [38].

Unexpectedly, birds with a higher natural parasitaemia showed a reduced RMR, a pattern also found in immunologically naive canaries when experimentally infected with *Plasmodium* [39]. As the nocturnal oxygen consumption in most birds in our study showed marked fluctuations during the migration period, it is unclear whether the lower RMR in the high-parasitaemia group was a result of hypometabolism *per se* or was a consequence of reduced nocturnal restlessness, a behaviour well known in migrating birds [34].

Also in contrast to our expectations, both MMR and endurance times were not affected by infection. Both are fundamentally coupled to the internal oxygen transport capacity and, therefore, we argue that a low parasitaemia does not greatly reduce oxygen transport or uptake, and any minor effect of infection on oxygen transport was greatly outweighed by inter-individual variability across all life-history stages examined.

### (b) Phenotypic changes across life-history stages

We found a striking seasonal pattern in RMR and MMR, with both increasing considerably towards the migration period. Birds preparing for migration had higher BMRs [10], higher aerobic capacity and could sustain exercise longer. Importantly, this pattern was shared by experimentally treated and control birds as well as wild birds.

Higher metabolic rates require higher oxygen supply to the mitochondria, which is facilitated by higher total haemoglobin content of blood (figures 2 and 3) or likewise by elevated haematocrit [35]. In parallel with the shared increases in aerobic capacity in the pre-migratory phase, non-infected and infected hosts displayed significant increases in accumulated fuel (in the form of body fat) and increased breast muscles at similar rates leading up to this period.

### (c) Implications for migration ecology of hosts and blood parasite infections

Because we found infected and uninfected migratory birds to be indistinguishable in MMRs and exercise endurance and to share morphological adjustments commensurate with migratory preparation, we conclude that low-parasitaemia birds will have the same migratory capacity as uninfected individuals. Assuming flight speed is physiologically constrained by aerobic capacity, our results suggest that chronically infected migrants are able to fly at speeds similar to uninfected individuals and we do not expect differences in the duration of flight bouts. There is ambiguous evidence for haematozoan infections to affect migration in passerines: blood parasites can delay arrival on the breeding grounds of migrants [40] or they have no effect on arrival times [41]. However, arrival might mainly be determined by factors other than flight speed because migrants usually spend more than 80% of total migration time at stopover sites [42]. Thus, infected and uninfected individuals can still differ in the timing of migration if, e.g. the efficiency of fundamental stopover behaviour like foraging and fuelling is hampered by the parasite and results in a later departure in infected birds than their non-infected counterparts.

Even if chronically infected birds arrive later, they still have the physiological capacity to migrate successfully over long distances, thus, they are mobile reservoirs for infections of conspecifics and unrelated species at stopover and at breeding sites. The consequences of such haematozoan infections on immunologically naive hosts can vary markedly, ranging from those that are totally refractory to infection to others that are highly compromised [4,43].

## 5. Conclusion

Our study has advanced our understanding of the interaction between blood parasites and their migratory hosts. We have excluded one long-speculated effect of these parasites—namely that on aerobic performance. Thus, if infected migratory hosts differ from uninfected conspecifics in any phenological measure, e.g. departure or arrival times, clutch size, or reproduction success, other mechanisms besides parasite-induced changes in aerobic capacity must be involved.

## Supplementary Material

Phenotypic characterisation of birds and summary statistics

## Data accessibility

Data are available from Dryad: http://dx.doi.org/10.5061/dryad.k50s6 [44].
