# Supplementary material for: Low intensity blood parasite infections do not reduce the aerobic performance of migratory birds
Source: Proc Biol Sci. 2018 Jan 31;285(1871):20172307. doi: 10.1098/rspb.2017.2307 (PMC5805937; doi:10.1098/rspb.2017.2307)
Supplement: Phenotypic characterisation of birds and summary statistics [file rspb20172307supp1.pdf]

Electronic Supplementary Materials

Figure S1. Phenotypic changes in great reed warblers during the course of the experiment.

The temporal changes of subcutaneous fat stores and pectoral muscle size from the pre-inoculation to the late post-inoculation period in experimentally infected (n=16) and non-infected birds (n=16) are given as medians ± min/max. Fat scores are estimated in intervals from 0-8, muscle scores range from 0-3 according [1].

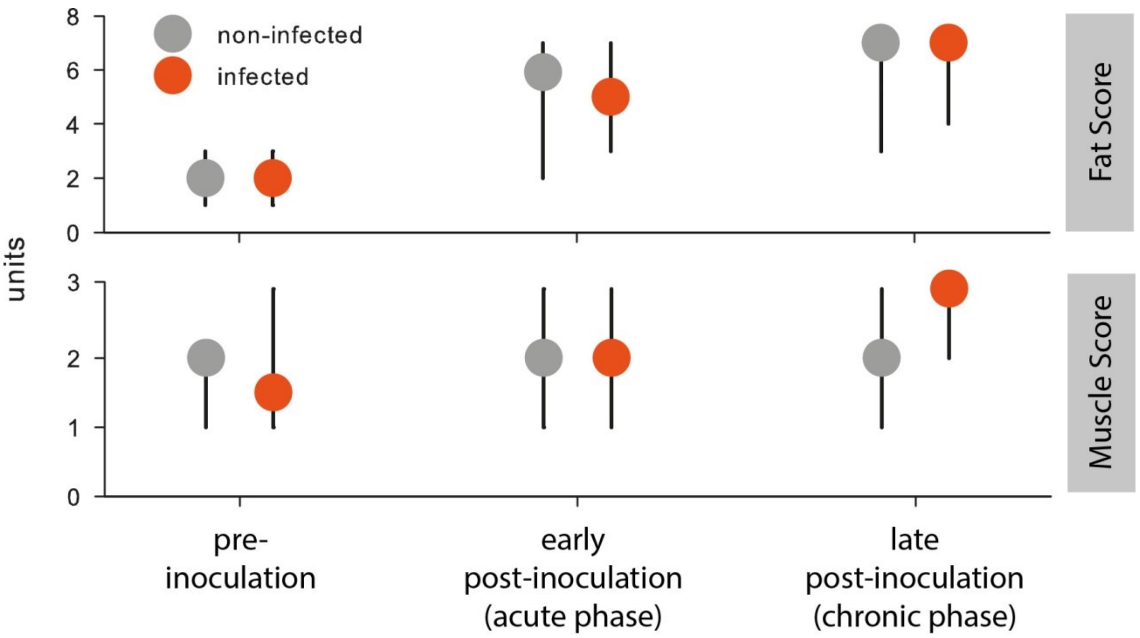

Figure S2. Phenotypic characterisation of free-living great reed warblers with different parasitaemia in consecutive life stages. Phenotypes are described by wing length (medians ± 25/75percentiles), body fat accumulation and the development of pectoral muscle (both medians ± min/max); infection status is given with and without low and high parasitaemia chronic infection. Birds had been captured during the late breeding, post-breeding and the migration period. Energy stores in the form of subcutaneous fat increased between late-breeding and migration periods.

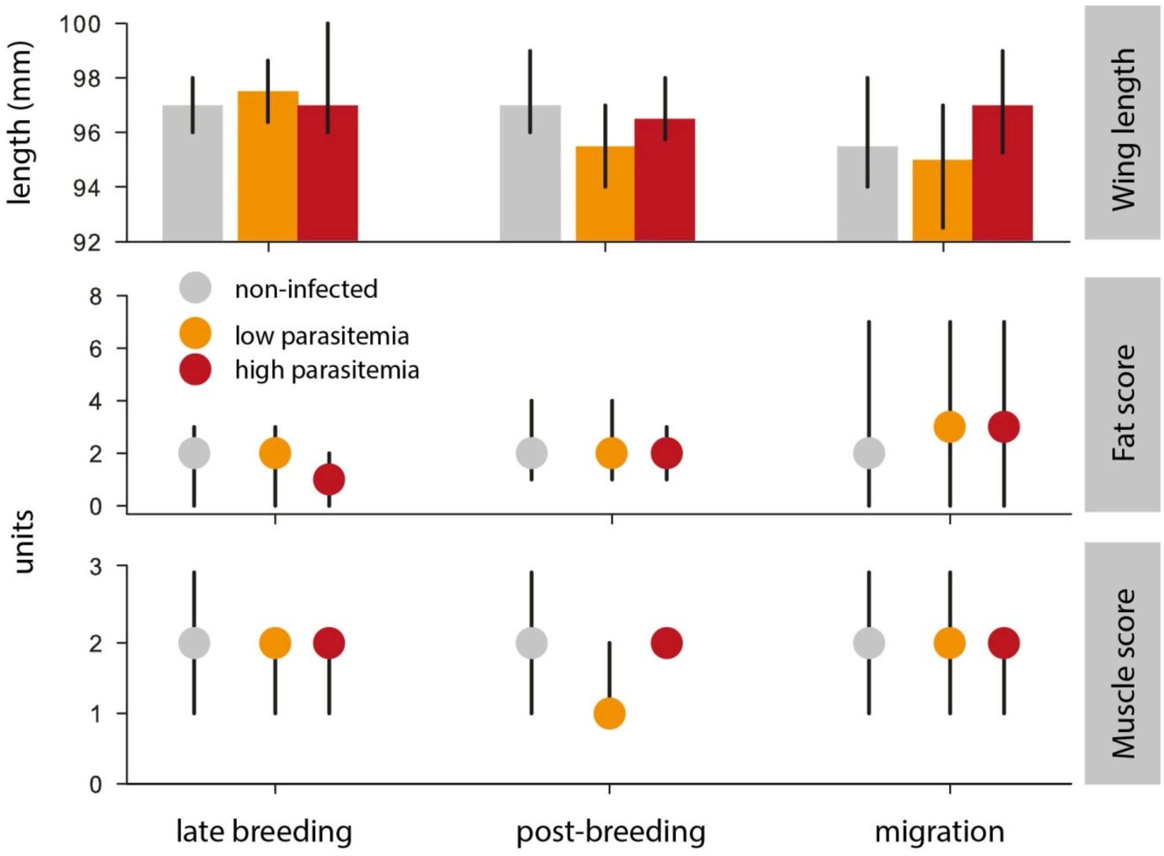

Table S1: Summary statistics for the experimental infection sub-study (n = 32 birds). Resting and maximal metabolic rates are oxygen consumption rates (ml min^{-1}), the endurance exercise is given as time to exhaustion (min), and haemoglobin concentration (mg ml^{-1}) refers to the haemoglobin content of whole blood. Changes in phenotype during the experiment are recorded in body mass (g), accumulation of fat and the development of pectoral muscle [1]. The factor infection refers to the experimental and the control group; the course of infection included pre-infection (reference level), acute infection and chronic infection phase. Metabolic rates, time to exhaustion and haemoglobin concentrations had been log transformed.

**Experimental infection**	Resting metabolic rate (RMR)			Maximal metabolic rate (MMR)			Time to exhaustion			Haemoglobin concentration		
	Coefficient ± SE	t	p	Coefficient ± SE	t	p	Coefficient ± SE	t	p	Coefficient ± SE	t	p
intercept	-1.343±0.21	-6.39	*	1.064±0.32	3.29	*	0.454±0.05	8.81	*	2.205±0.01	267.85	*
infection	-0.011±0.02	-0.48		-0.008±0.04	-0.20		-0.030±0.07	-0.42		-0.013±0.01	-1.21	
acute phase	0.027±0.03	1.00		0.182±0.05	3.82	*	0.129±0.07	1.75		0.016±0.01	1.72	
chronic phase	-0.019±0.03	-0.64		0.167±0.05	3.16	*	0.318±0.07	4.42	*	-0.005±0.01	-0.58	
log (body mass)	0.953±0.14	6.31	*	-0.013±0.22	-0.06							
infection × acute		0.01			0.30		0.082±0.10	0.83		-0.002±0.01	-0.19	
infection × chronic		1.40			0.23		-0.03±0.10	-0.29		0.026±0.01	2.07	
	Body mass			Fat score			Pectoral muscle score					
Intercept	28.70±1.26	22.81	*	1.923±0.34	5.60	*	1.615±0.17	9.54	*			
Infection	-0.88±1.69	-0.52		-0.236±0.46	-0.51		-0.053±0.23	-0.23				
acute phase	7.62±1.56	4.90	*	3.340±0.43	7.71	*	0.219±0.20	1.07				
chronic phase	12.33±1.37	8.99	*	4.38±0.42	10.37	*	0.69±0.20	3.46	*			
infection × acute phase	3.26±2.07	1.57		0.161±0.577	0.28		0.343±0.27	1.26				
infection × chronic phase	1.77±1.93	0.91		0.402±0.59	0.68		0.353±0.28	1.25				

P values: lmer function does not provide p values because, at present, it is unclear how to calculate the appropriate degrees of freedom in lme. We considered factors with absolute t values>2 significant at the p<0.05 level (*) following suggestions in [2]

Table S2: Summary statistics for the natural infection sub-study (n = 156 birds). Resting and maximal metabolic rates are oxygen consumption rates (ml min^{-1}), the endurance exercise is given as time to exhaustion (min), and haemoglobin concentration (mg ml^{-1}) refers to the haemoglobin content of whole blood. Phenotypes of birds were characterized with body mass (g), wing length (mm), the accumulation of fat and the development of pectoral muscle. Infection status and strength of infection are categorized as uninfected (zero parasitaemia, reference level), low parasitaemia (<0.2%) and high parasitaemia birds (0.2 to max. 4.0%). The study period includes three subsequent periods: the late breeding period (reference level), the post-breeding and the migration period. Metabolic rates, time to exhaustion and haemoglobin concentrations had been log transformed.

**Natural infection**	Resting metabolic rate (RMR)			Maximal metabolic rate (MMR)			Time to exhaustion			Haemoglobin concentration		
	Coefficient ± SE	t	p	Coefficient ± SE	t	p	Coefficient ± SE	t	p	Coefficient ± SE	t	p
Intercept	-1.576±0.19	-8.46	0.001	0.369±0.28	1.34	0.18	0.420±0.02	21.7	0.001	2.178±0.005	420.90	0.001
Low parasitaemia	-0.016±0.01	-1.10	0.27	0.002±0.02	0.10	0.92	0.038±0.03	1.42	0.16	-0.001±0.007	-0.07	0.95
High parasitaemia	-0.037±0.02	-2.11	0.04	0.018±0.03	0.73	0.47	-0.033±0.03	-0.99	0.32	0.001±0.009	0.04	0.97
Post breeding period	0.031±0.01	2.11	0.04	0.111±0.02	5.21	0.001	0.075±0.03	2.72	0.007	0.015±0.007	2.07	0.04
Migration period	0.061±0.02	3.78	0.001	0.036±0.02	1.60	0.11	0.207±0.03	7.11	0.001	0.030±0.008	4.03	0.001
Log (body mass)	1.119±0.13	8.69	0.001	0.456±0.19	2.39	0.02	na			na		
	Body mass			Fat score			Pectoral muscle score			Wing length		
Intercept	27.81±0.47	59.11	0.001	1.555±0.19	8.36	0.001	1.535±0.07	20.9	0.001	97.11±0.29	335.76	0.001
Low parasitaemia	0.13±0.66	0.19	0.85	0.136±0.26	0.53	0.60	0.111±0.10	1.11	0.27	-0.65±0.40	-1.62	0.11
High parasitaemia	-0.21±0.81	-0.26	0.79	-0.194±0.31	-0.62	0.54	0.149±0.12	1.21	0.23	0.30±0.49	0.61	0.54
Post breeding period	0.38±0.68	0.56	0.58	0.612±0.27	2.30	0.023	0.048±0.10	0.45	0.65	-0.03±0.41	-0.8	0.94
Migration period	2.17±0.71	3.04	0.003	1.455±0.28	5.28	0.001	0.352±0.11	3.23	0.002	-1.23±0.43	-2.82	0.005

[1] Eck S, Fiebig J, Fiedler W, Heynen I, Nicolai B, Töpfer T, van den Elzen R, Winkler R, Woog F. 2011 *Measuring birds*. Wilhelmshaven: Deutsche Ornithologen-Gesellschaft.
[2] Baayen RH. 2008 Analyzing linguistic data : a practical introduction to statistics using R. Cambridge: Cambridge University Press.
